# Developing a robust model to predict depth of anesthesia from single channel EEG signal

**DOI:** 10.1007/s13246-022-01145-z

**Published:** 2022-07-05

**Authors:** Iman Alsafy, Mohammed Diykh

**Affiliations:** 1 College of Education for Pure Sciences, University of Thi-Qar, Nasiriyah, Iraq; 2 USQ College, University of Southern Queensland, Toowoomba, QLD 4350 Australia; 3 Information and Communication Technology Research Group, Scientific Research Centre, Al-Ayen University, Nasiriyah, Iraq

**Keywords:** BIS, HDE, EEG, DoA, CGDA, Sliding window, Statistical metrics

## Abstract

Monitoring depth of anaesthesia (DoA) from electroencephalograph (EEG) signals is an ongoing challenge for anaesthesiologists. In this study, we propose an intelligence model that predicts the DoA from a single channel electroencephalograph (EEG) signal. A segmentation technique based on a sliding window is employed to partition EEG signals. Hierarchical dispersion entropy (HDE) is applied to each EEG segment. A set of features is extracted from each EEG segment. The extracted features are investigated using a community graph detection approach (CGDA), and the most relevant features are selected to trace the DoA. The proposed model, based on HDE coupled with CGDA, is evaluated in term of BIS index using several statistical metrics such Q-Q plot, regression, and correlation coefficients. In addition, the proposed model is evaluated against the BIS index in the case of the poor signal quality. The results demonstrated that the proposed model showed an earlier reaction compared with the BIS index when patient’s state transits from deep anaesthesia to moderate anaesthesia in the case of poor signal quality. The highest Pearson correlation coefficient obtained by the proposed is 0.96.

## Introduction

Monitoring the depth of anaesthesia is a vital element that must be carried out carefully during surgical procedures because any error can cause intraoperative awareness, in which patients experience unexpected recall of events taking place during surgery [1]. Neurologists normally monitor the DoA to deliver an adequate amount of drug to patients, because inadequate depth can increase postoperative risks [2, 3]. Medical reports have shown that incidents of awareness are higher among cardiac surgery (1.1–1.5%), caesarean Sect. (0.4%), and trauma surgery (11–43%) [4]. A number of approaches have been developed to assess the DoA such as clinical signs based methods (blood pressure, pulse, respiratory rate, oxygen immersion, and sweating), heart rate variability based approaches, isolated forearm technique (IFT), and lower oesophageal contractility (LOC). However, those method are not always adequate to deliver the right amount of drug to patients and could not obtain the desired accuracy of DoA [3–7]. For example, clinical studies have proved that some patients who endured awareness during surgery, did not show any abnormalities in blood pressure, pulse, and respiratory rate [8–11]. Which makes the interpretation of these signs very difficult for clinicians. LOC is not accurate enough for detecting of DoA when the main anaesthetic agent is used [2]. With the IFT technique, anaesthesiologists ask patients to move their hands to check the level of consciousness [12–15]. However, some patients are unable to move their figures due to hearing issues.

Medical studies have found that anaesthetic drugs affect the nervous system and the electrical brain activities change correspondingly with the DoA during surgery [16, 17]. Accordingly, many commercial models for monitoring the DoA have been developed based on electroencephalograms EEG signals. Currently Bispectral Index (BIS) is considered the standard device for prediction the DoA [22–25]. Although the BIS has been proven to be effective in practice, it has limitations in many aspects [18–21]. For example, it does not work effectively with all anaesthetic drugs, is not accurate across patients, and is being delayed [26, 27]. In addition, the BIS and other developed devices have shown a long-time delay to reflect a change in a state of consciousness [28]. To address these issues numerous models have been developed to trace the DoA over the recent years. For example, Shih-Jui Chen et a1. [29] designed an EEG based model to characterize anesthetic states. EEG signals were decomposed into a set of internal mode functions (IMFs) using an empirical mode decomposition. In that study, Fast Fourier Transform (FFT) and Hilbert Transform (HT) were integrated and applied to each IMF. Probability distributions of all frequencies were generated from two groups of patients and used to trace the DoA. Their results showed that frequencies of IMF can reflect the DOA. Vidhya et al., [11] analyzed anesthetic EEG signals to design an accurate DOA model. A total of 54 subjects, 37 adults and 17 children, were involved in that study. A simultaneous functional near-infrared spectroscopy was employed to analyse EEG signals to monitor the DOA. Zoughi et a1., [30] employed a wavelet transform technique to analyse EEG signals of patients. An energy-based entropy feature was extracted from wavelet coefficients, and used as a statistical attribute to monitor the DoA. To validate their suggested index, a total of 22 EEG recordings were used in the evaluation phase. Their obtained results showed that there was a high correlation between the suggested index and BIS during anesthetic states. Bauerle et a1., [28] used a statistical approach to analyse EEG signals. The statistical analysis was performed using SPSS software. In that study, different entropy features were calculated to investigate the correlation between a subject’s state and the DoA. They showed that approximate entropy, Shannon entropy, and Lempel Ziv decreased gradually during the depth of anesthesia.

Benzy et a1., [31] integrated a wavelet transform with a neural network model to assess the depth of anesthesia. Wavelet coefficients were investigated and then a list of features was extracted to trace the DoA. Shalbaf et al., [32] suggested an automatic method for assessing depth of anesthesia. Two features: sample entropy and permutation entropy, were extracted from EEG signals to quantify the amount of the DoA. The extracted features were transferred to an artificial neural network model. They obtained an accuracy of 88%. Halder et al. [33] investigated the use of Wada test to predict the DoA from 7 subjects. A power spectral density, functional connectivity, and measures of signal diversity were adopted to assess the DoA. Moca et al., [34] integrated a time-domain approach with a multi-layer perceptron network to trace DOA levels. Diykh et al., [35] recognised the states of anesthesia using a complex network approach. EEG signals were segmented, and statistical features were extracted. The extracted features were mapped into graphs. Spectral based wavelet graphs coefficients were investigated and used to design a DoA index.

In recent days, deep learning techniques have been become more popular way to analyse EEG signals. For example, Chowdhury et al. [23], proposed a deep learning model to assess the DoA. A total of 50 subjects were involved in that study. Two signals: Electrocardiography (ECG), and photoplethysmogram (PPG), were used to assess the proposed index of DoA. Each single channel signal was converted into an image form. The produced images were fed into the proposed deep learning model. Li, et al., [27] combined long short-term memory and a sparse denoising autoencoder to monitor the depth of anesthesia. EEG signals were filtered and then several features including sample entropy, permutation entropy, spectra, and alpha ratio were extracted from the EEG signals. The extracted attributes were fed to the long short-term memory and a sparse denoising autoencoder. AlMeer et al. [36], designed a deep learning model to track the DoA. They found that linear features were correlated with the BIS index.

With respect to the work done by the previous studies to improve DoA monitoring, this study aims to develop a robust BIS prediction model. Although there have been studies that forecasted BIS values, the developed model in this study differs from previous studies in two aspects. First, the association between the EEG decomposition level with the DoA is investigated, and secondly, we designed a feature extractor approach based on the HDE method to take advantage of the fast calculation and good stability, resulting in finding that the HDE method has a high potential in DoA monitoring. In this research article a new methodology for the DoA monitoring based on the hierarchical dispersion entropy (HDE) approach is proposed. The main contributions of this paper are reflected as follows: (1) Introduce a new scheme for an advanced DoA monitoring system involving HDE, features selection model based on CGDA; (2) Propose Hierarchical Diffusion Entropy (HDE) for extracting efficient characteristics from EEG data; (3) Utilise statistical metrics to eliminate the most redundant information from EEG data.

## Methodology

**Fig. 1:**
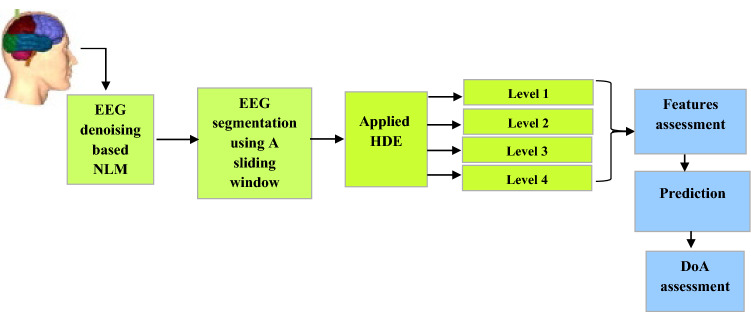
The proposed framework of DoA monitoring

In this study, a robust model is developed to monitor DoA based on Hierarchical Diffusion Entropy (HDE). Figure 1 describes the proposed methodology to assess the DoA. The original EEG signals are denoised using an improved nonlocal mean method. Then, the denoised EEG signals are partitioned into intervals using a sliding window technique. The EEG signals are decomposed into four levels. HDE is calculated from each EEG segment. The extracted features are tested using a community graph detection approach (CGDA) to select the most powerful features to represent EEG signals. The proposed model is evaluated against the BIS using regression, correlation coefficient, and Q-Q plot. The obtained features showed a high correlation between the proposed model and the BIS when a patient’s state transits from a light anesthesia to a deep anesthesia.

### EEG signal de-noising and sample selecting

**Fig. 2:**
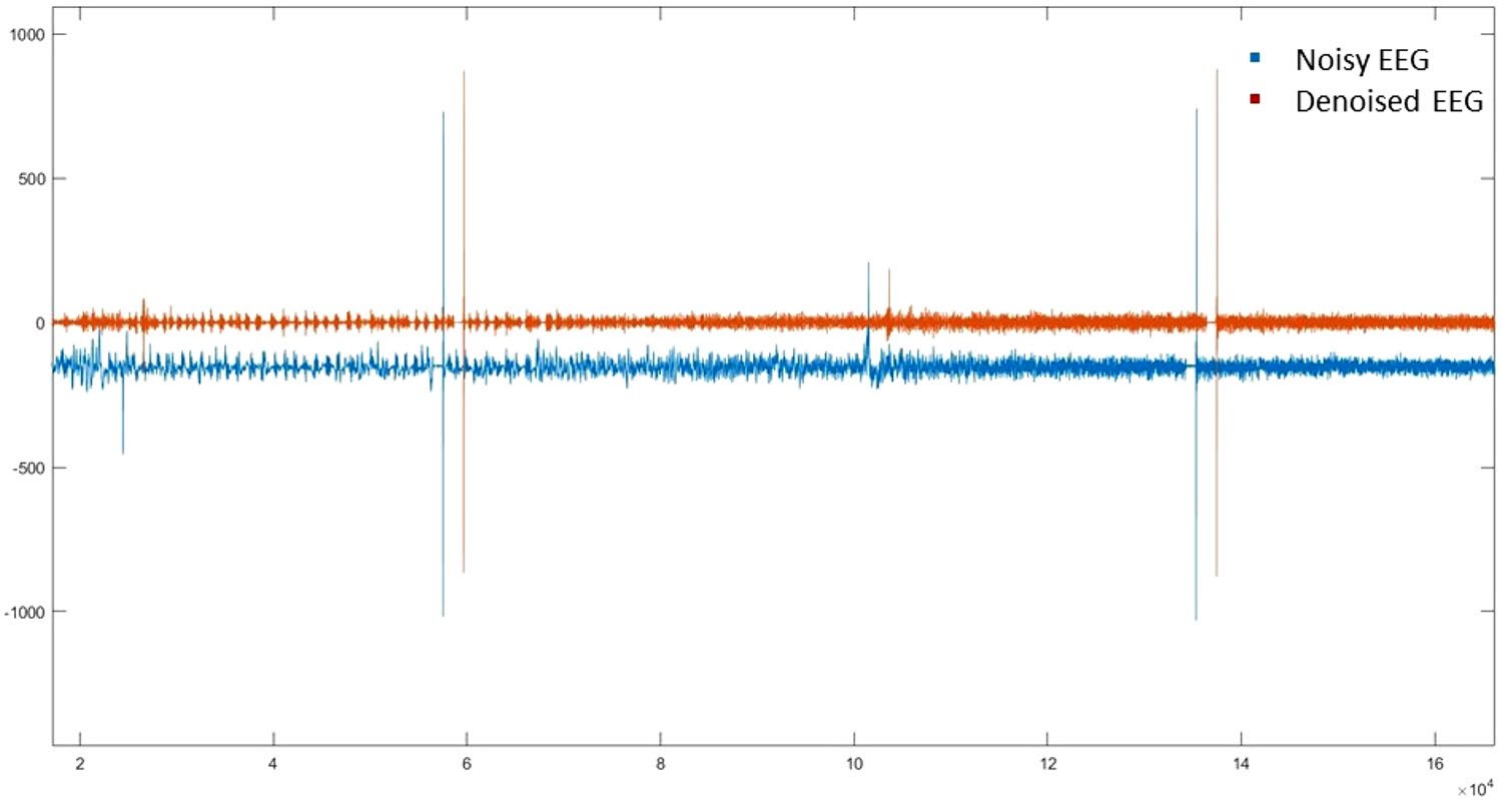
A comparison between the noisy and denoised EEG signals

The captured EEG signals were filtered to remove noises including electrocardiogram (ECG), EMG (muscle stimulation) noise, and noise generated from devices utilized in the operating room, such as the interference of wires energy, and poor fixation of the EEG electrodes. In this study, an improved nonlocal mean method (NLM) was adopted to filter EEG signals. The improved NLM method was combined with a wavelet transform (WT) method. The WT was employed to decompose the aesthetic EEG signals into different levels of Wavelet coefficients. Then the NLM was applied to the Wavelet coefficients. The filtered Wavelet coefficients were processed to reconstruct the noiseless EEG signals. For details of this approach, please refer to our previous research work [37, 38]. Figure 2 shows denoised and noisy EEG signals.

To accurately evaluate the performance of the proposed model against different anesthetic states, the selected EEG sample must balance both the anesthetic states and awake states. For instance, at the beginning, the BIS monitor often showed a series of unknown values or stayed at the value 97.7 without change in its value. In addition, it is noticed that the SQI index values in some cases were lower than 15 at the beginning. However, we selected the samples in which SQI index values were high enough and the BIS values and raw EEG data were excellent at the beginning for the performance evaluation. As a result, a total of 18 subjects were selected in this study. The ID Nos. of those subjects were 2–8, 11, 13, 18, 19, 20, 21, 22, 24, 25, 29 and 30.

**Fig. 3:**
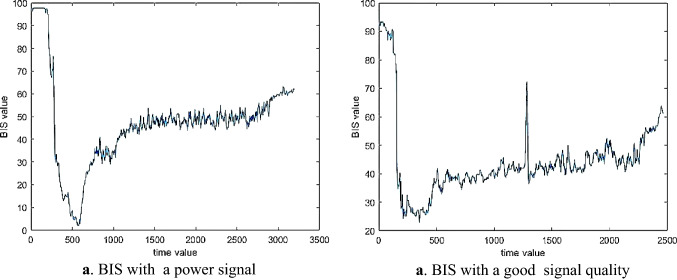
An example of a good signal quality and poor signals quality

We also assessed the performance of the proposed method in poor signal quality cases. That means when SQI was lower than 15, the BIS index could not display valid values on the screen. In this study, the subjects 14, 12, 17 and 32 were selected as a sample of poor signal quality. Figure 3 shows an example of EEG signals with a high signal quality and a low signal quality.

### Segmentation

**Fig. 4:**
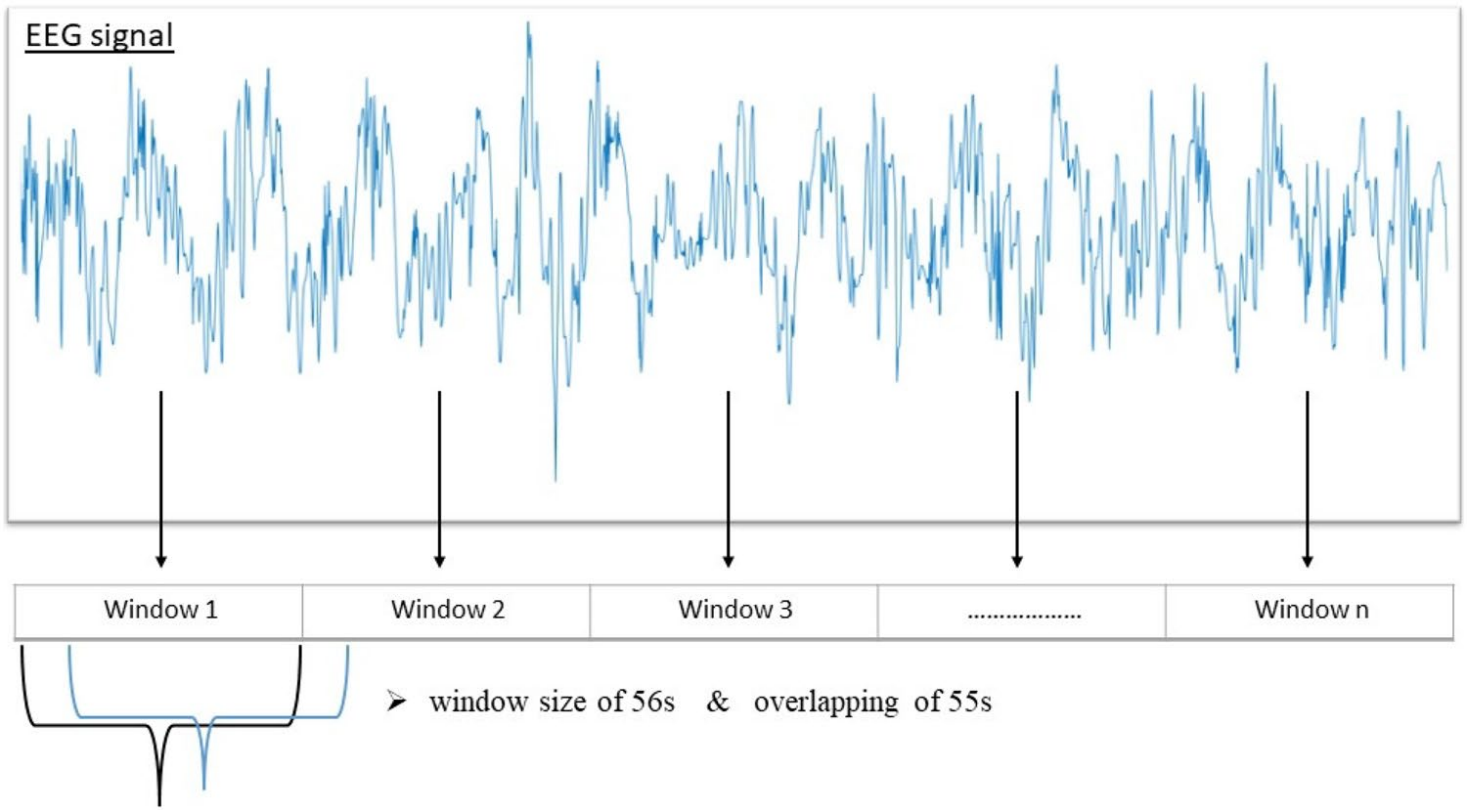
an example of an EEG signal is being segmented using window technique

To calculate hierarchical dispersion entropy, a sliding window technique is applied to segment EEG signals. Figure 4 depicts the segmentation technique. A window size of 56 s was selected in this study with an overlap 55 s. Based on our previous studies [35], the calculations based on the window size of 56 s with the overlapping of 55 s gave satisfactory DoA assessment results. Suppose $X$ is an EEG signal of $n$ datapoints, $X = \left\{ {x_{1} , x_{2} , x_{3} , \ldots \ldots , x_{n} } \right\}$. The EEG signal $X$ was divided into $M$ overlapped windows where $M = \left\{ {w_{1} , w_{2} , \ldots .., w_{M} } \right\}$, where $w_{M} = \left\{ {x_{1} , x_{2} , \ldots ., x_{k} } \right\}$ . As a result, the EEG signal is partitioned into $M$ segments, with each window containing 6720 datapoints. For further investigation, different window sizes are also tested, and all obtained results are reported. More detail is seen in the Experimental results section.

### EEG signals analysis based on hierarchical diffusion entropy (HDE)

#### Diffusion entropy (DE)

In this study, the diffusion entropy was utilized to assess the DoA from EEG signals. In particular, the DE is calculated based on the following steps [38–42].

1. Suppose $X = \left\{ {x_{1} , x_{2} , x_{3} , x_{4} \ldots \ldots .., x_{n} } \right\}$ is an EEG signal, where $n$ is the number of samples in $X$. A normal distribution function was employed to transfer each sample into a new sequence $Y$ where $Y = \left\{ {y_{1} , y_{2} , y_{3} , y_{4} \ldots \ldots .., y_{n} } \right\}$, $Y \in \left( {0,1} \right).$
  1 $$Y_{i} = \frac{1}{{\sigma \sqrt {2\pi } }}\mathop \smallint \limits_{ - \infty }^{{x_{i} }} \exp \frac{{ - (k - u)^{2} }}{{2\sigma^{2} }}$$
  In Eq. (1), $u\,and\,\sigma$ is refers to the deviation.
2. Based on a linear transformation, $z^{\left( d \right)} = int\left( {dy_{i} + 0.5} \right)$ is calculated, where $int$ is a rounding function and $d$ is a group number. Each $y$ is assigned to an integer group ranged in $\left[ {1,2, \ldots ., d} \right)$.
3. The embedding vector $z_{i}^{{\left( {n,d} \right)}}$ is computed using the following formula to form each embedding vector $z_{i}^{{\left( {n,d} \right)}}$ with embedding dimension $n$ and time delay $d$
  2 $$Z_{i}^{{\left( {n,d} \right)}} \; = \;(z_{i}^{{\left( d \right)}} ,~\;z_{{i + c}}^{{\left( d \right)}} ,~ \ldots ,~z_{{i + \left( {n - 1} \right)c}}^{{\left( d \right)}}$$
  $$where\,i = 1,2,3,{ } \ldots \ldots ,{ }M - \left( {n - 1} \right)d$$
  Each time series $z_{i}^{{\left( {n,d} \right)}}$ is mapped into a dispersion pattern $\pi_{{r_{1} ,{ }r_{2} ,{ } \ldots .,{ }r_{{n{ }}} }}$ where $z_{i}^{d} = r_{1} ,{ }z_{i + c}^{d} = r_{2} ,{ } \ldots ..,{ }z_{{i + \left( {n - 1} \right)c}}^{d} = r_{n - 1}$, where $d^{n}$ denotes to the total number of dispersion patterns.The relative frequency dispersion pattern $d^{n}$ is calculated based on the following formula
  3 $$L\left( {\pi_{{r_{1} , r_{2} , \ldots ., r_{n - 1 } }} } \right) = \frac{{Num(\pi_{{r_{1} , r_{2} , \ldots ., r_{n - 1 } )}} }}{{M - \left( {n - 1} \right)c}}$$
  $L\left( {\pi_{{r_{1} ,{ }r_{2} ,{ } \ldots .,{ }r_{{n - 1{ }}} }} } \right)$ indicates to the number of dispersion patterns $,{ }$ $Z_{i}^{{\left( {n,d} \right)}}$ is divided by the total number of embedding data with embedding dimension $n$.
4. Based on the definition of entropy, the dispersion entropy is defined as:
  4 $$En\left( {x,n,d,c} \right) = - \mathop \sum \limits_{\pi = 1}^{{d^{n} }} L\left( {\pi_{{r_{1} , r_{2} , \ldots ., r_{n - 1 } }} } \right)\ln \left( {L\left( {\pi_{{r_{1} , r_{2} , \ldots ., r_{n - 1 } }} } \right)} \right)$$

#### Hierarchical diffusion entropy (HDE)

Dispersion entropy can analyse a certain frequency band of signals, however, one of the deficiencies of dispersion entropy is that it disregards the full frequency bands. To overcome this limitation, we have adopted the HDE method to analyse the HAR data more accurately. In essence, the HDE algorithm steps can be described as follows [38–42]:

1. Suppose we have a time series $X = \left\{ {x_{1} , x_{2} , x_{3} , x_{4} \ldots \ldots .., x_{n} } \right\}$ where $n$ is the number of samples in a signal $X$. The average of operator $G_{i}$ is calculated as follows:
  5 $$G_{i} = \frac{{X\left( i \right) \mp x\left( {i + 1} \right)}}{2}$$
  In Eq. (5), $i = 1,2,3,\,\ldots ., N - 1$, and $N = 2^{n}$, $n$ is a positive integer number.
2. $i = 1\,or\,0$, when $i = 0,$ the symbol gets positive in Eq. (5), or negative when $i = 1$.
3. The layer matrix of the operator $G_{j}^{k} \left( {i\; = \;0\;or\;1} \right)$ is defined as
  6 $$G_{j}^{k} = \left\{ {\begin{array}{*{20}c} \frac{1}{2} \\ 0 \\ 0 \\ \end{array} \begin{array}{*{20}c} {\frac{ - 1}{2}} \\ 0 \\ 0 \\ \end{array} \begin{array}{*{20}c} 0 \\ \frac{1}{2} \\ 0 \\ \end{array} \begin{array}{*{20}c} 0 \\ {\frac{ - 1}{2}} \\ 0 \\ \end{array} \begin{array}{*{20}c} { \ldots \ldots .} \\ { \ldots \ldots } \\ { \ldots \ldots .} \\ \end{array} \begin{array}{*{20}c} 0 \\ 0 \\ \frac{1}{2} \\ \end{array} \begin{array}{*{20}c} 0 \\ 0 \\ {\frac{ - 1}{2}} \\ \end{array} } \right\}$$
4. A vector $E$ is constructed where $E_{n} = 0{ }or{ }1$, the integer $v$ is defined as
  7 $$v = \mathop \sum \limits_{i = 1}^{n} 2^{n - 1}$$
  Based on the Eq. (7), $E$ is a non-negative integer and the vector $\left[ {v_{1} ,{ }v_{2} ,{ }v_{3} ,{ } \ldots .,v_{n} } \right]$ equivalent to the $v$.
5. Each node of each layer that defines the time series $X$ is
  8 $$X_{k, v}^{ } = G_{{e_{1} }}^{ } *G_{{e_{2} }}^{ } * \ldots \ldots \ldots *G_{{e_{n} }}^{ } \left( x \right)$$

**Fig. 5:**
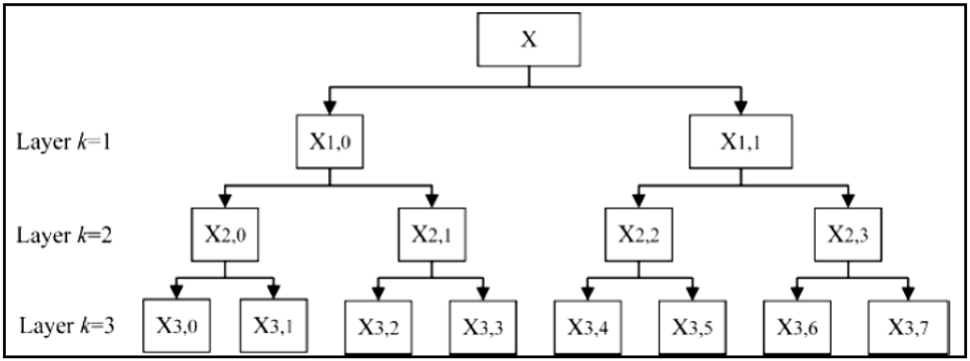
Flowchart of HDE with three layers

In Eq. (8), $k$ refers to the number of the layers in hierarchical segmentation, and DE is acquired by calculating the entropy for each hierarchical component HDE. Figure 5 shows the analysis stages of the HDE method with its three-layer step.

#### Parameter selection for HDE method

Four parameters were chosen carefully in this paper to calculate HDE: embedded dimension, class number, time delay and hierarchical layers.

- We set the embedded dimension $n$ to 3. Based on the study in [42], it was found that if $n$ was too large, more data length was needed.
- We set the time delay $d$ to 1. The previous study in [42] showed that when $d > 1$ more significant data regarding the frequency can be lost.
- We set the number of hierarchical layers $k$ to 3. We found that if $k > 3$ the calculation efficiency and sample points involved in each level were increased. However, if $k < 3$ the frequency band of signal was not carefully divided, and the hierarchical components become insufficient [42].
- Class number was set to 5. We found that when $class number$ was too small, different features were categorized as a one class, while when it was too large, similar features were classified in different classes [42].

### Feature selection based on community graph detection and normalization

We applied the HDE to EEG segment to capture the desired features to assess the DoA. The HDE was calculated from four level. It was found that the HDE at level 2 across 22 subjects increased during the awake state, when the whole brain is active. However, it degraded during the anaesthetic states.

To select the most important features from EEG segments, the extracted HDE from EEG segments were analysed using a graph-based community detection technique (GBCD) [43–46]. We selected only the most significant features which can reveal the changes in EEG waves during the DoA. It was also found that when the EEG signal transits from moderate to deep anaesthesia, only features of level 2 reflect those changes in EEG signals including. We proposed GBCD to select a set of important features to represent EEG data. The feature selection method proposed in this paper works according to following steps.

- Firstly, the entire feature set is transferred into a weighted graph.
- Secondly, GBCD is used to partition the features into several groups.
- Finally, a node centrality metric is employed to select the final subset of features.

**Fig. 6:**
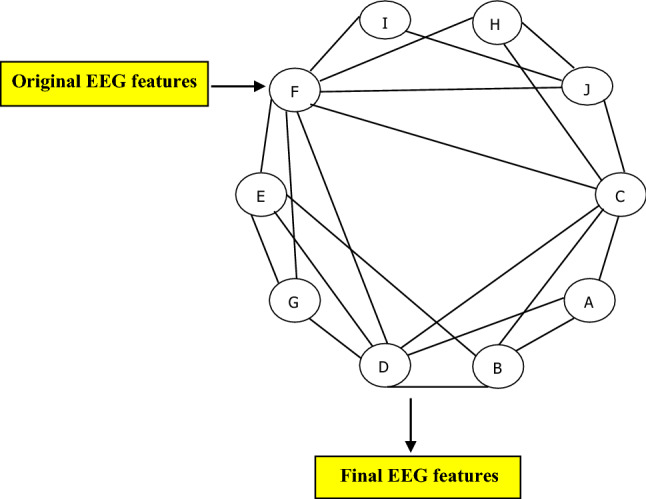
A vector of EEG features is transferred into a graph

The extracted features set was mapped as a weighted graph $G = \left( {N,{ }E,{ }W} \right)$, where $N$ represents a set of original features, $E$ is the edges of the graph, and $W$ refers to the degree of similarities among nodes. Different metrics are available to calculate nodes similarities. In this paper, we examined three metrics namely Pearson correlation coefficient, Euclidean distance, and Pearson Product Moment Correlation Coefficient (PPMCC). Based on the obtained results, we found that PPMCC gave more accurate results. Figure 6 shows EEG features are being transferred into a graph.

**Fig. 7:**
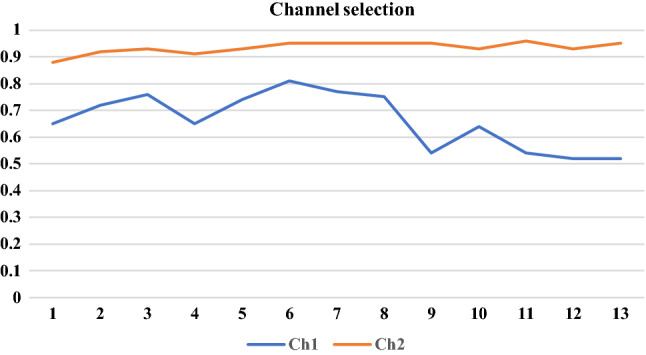
Channel selection based on correlation coefficient

The similarity between two features based on PPMCC can be calculated as follow:

9 $$Q_{i,j} = \left[ {\frac{{\mathop \sum \nolimits_{S} \left( {p_{i } - \overline{p}_{i } } \right)\left( {p_{j } - \overline{p}_{j } } \right)}}{{\sqrt {\mathop \sum \nolimits_{S} (p_{i } - \overline{p}_{i } )^{2} } \sqrt {\mathop \sum \nolimits_{s} (p_{i } - (\overline{p}_{i } )^{2} } }}} \right]$$

where $p_{{i{ }}}$ and $p_{{j{ }}}$ represent the features sets, $\overline{p}_{{i{ }}}$ and $\overline{p}_{{j{ }}}$ refers to the mean values of sets $p_{{i{ }}}$ and $p_{{j{ }}}$ divided by $S$ samples. The outputs of $Q_{i,j}$ range between $\left[ {1,0} \right]$, where 1 indicates the pair $p_{{i{ }}}$ and $p_{{j{ }}}$ of is completely similar while 0 denotes the pair $p_{{i{ }}}$ and $p_{{j{ }}}$ are dissimilar. In some cases, it is found that some values of $Q_{ }$ are very close to each other. To tackle this issue, a nonlinear normalisation method called SoftMax scaling is utilised to normalise the values of $Q_{ }$ into the range [1–0] based on the following formula:

10 $$\tilde{Q}_{i,j} = \frac{1}{{1 + \exp \left( { - \frac{{Q_{i,j} - \overline{Q}}}{\rho }} \right)}}$$

where $Q_{i,j}$ is the normalised value, while $\rho$ is the mean and variance of the similarity values. Figure 7 shows the graph construction process.

In this paper, a community detection technique is used, based on the feature selection method to eliminate the redundant features. The community detection technique aims to categorise the graph nodes into different communities based on their similarities. Each community includes characteristics that are similar to each other. In this paper, we adopted the Louvain community detection technique to partition graph nodes into clusters. We adopted the Louvain community detection technique because it is simple, and easy to apply. The performance of the Louvain community detection algorithm is improved by eliminating edges associated with weights lower than a predefined threshold $\sigma$. The value of $\sigma$ is between 1 and 0. We made a thorough analysis to select the value of $\sigma$. As a result, we set $\sigma$ to 0.5. The Louvain community detection algorithm works based on three steps:

1. The graph modularity $q$ is maximised by assigning each node $i$ in a graph $G$ to a community according based on the following formula
  11 $$q = \frac{{\mathop \sum \nolimits_{n} + k_{i}^{n} }}{2j} - \left( {\frac{{\mathop \sum \nolimits_{\mathop n\limits } + k_{i}^{ } }}{2j}} \right)^{2} - \frac{{\mathop \sum \nolimits_{n} }}{2j} - \left( {\frac{{\mathop \sum \nolimits_{\mathop n\limits } }}{2j}} \right)^{2} - \left( {\frac{{\mathop \sum \nolimits_{n} }}{2j}} \right)$$
  where $\mathop \sum \limits_{n} { }$ is the total sum of nodes weight in $n$ community, $\mathop \sum \limits_{\mathop n\limits } { }$ refers to the total sum of nodes weights incidental to nodes in $n$, $k_{i}^{ }$ denotes to the total sum of nodes weights incident to node $i$, $k_{i}^{n}$ refers to the total sum of nodes weights from $i$ to nodes in $n$, and $j$ is the total sum of nodes weights in graph.
2. Generate a new graph that contains the nodes of all the communities attained in step 1.
3. Step 1,2 is repeated until a significant improvement of the graph modularity is achieved.

The integration of node centrality is calculated to find the most influential features in each cluster as well as to remove the poor features. The influential degree $f_{i}$ ${\text{inf}}\left( {f_{i} } \right)$ is expressed as

12 $$\inf \left( {f_{i} } \right) = Lp\left( {f_{i} } \right)x Nor\left( {S, f_{i} } \right)$$

13 $$Nor\left( {S, f_{i} } \right) = \frac{1}{\left| S \right|}\mathop \sum \limits_{j = 1}^{\left| S \right|} (B_{ij} - \overline{B}_{i} )$$

where $Lp\left( {f_{i} } \right)$ refers to the Laplacian centrality of feature $f_{i} .$ $Nor$ indicates to the normalised variance of feature $f_{i}$, while $\left| S \right|$ is the total number of patterns, $B_{ij}$ is the value of feature $f_{i}$ for pattern $j$. After the influential degree is calculated for each feature, a threshold $\varphi$ is employed to remove the features that their influential values are less than $\varphi$. Then, the clustered graph is recreated. The procedure is repeated until no node with influential value less than $\varphi$ is observed. In this paper, we found that not all features reflected the changes in EEG during the DoA. More details are discussed in the next section.

## Experimental results

In this section, the main findings were discussed using several statistical metrics. Root Mean Square Error (RMSE) and accuracy, sensitivity, specificity, Q-Q, regression, and correlation coefficients were used to evaluate the performance of the proposed model. The extracted features were analysed to find the best set of features to trace the DoA. The EEG data from two EEG channels (Ch1, and Ch2) were tested separately, and our finding showed that Ch 2 gave satisfactory results. MATLAB R2020a was utilised to implement the proposed model. Signal processing toolbox was adopted in the implementation.

### Anesthetic EEG data

Ethical approval was granted by the Human Research Ethics Committee at the University of Southern Queensland (No: H09REA029) and Darling Downs Health Service District Human Research Ethics Committee (No: TDDHSD HREC 2009/016), Australia. The total number of patients involved in this study was 37 adult subjects including 15 females and 22 males. Their demography information and anesthesia type are presented in Table 1. To record EEG signals, four adhesive forehead Quatro electrodes were attached to each patient. The collected EEG data were transferred and stored in a desktop computer for the offline analysis. The acquired EEG data included BIS index, EEG signals, monitoring error log, and the real time log. The obtained EEG signals were sampled at frequency of 128 Hz. A MATLAB code was designed to convert the EEG data from ASCII format to a signed number form.

**Table 1:** Patients’ characteristics

Demography information	
Gender (M/F)	12/15
Age (years)	22–35
Weight	55–150
*Anesthesia type*	
Midazolam (mg)	2–5
Alfentanil (mg)	500, 750, 1000
Propofo l (mg)	90–200
Parecoxib (mg)	40
Fentany1 (μg	100–150

EEG recordings which were acquired from two channels Ch1, and Ch2, were used in the simulation results. To select the optimal features to monitor the DoA, the extracted features from Ch1, and Ch2, were tested separately. The Pearson correlation coefficient was utilized to calculate the strength of the linear relationship between EEG channels and the BIS index. Its values ranged between (1-,1) where 1- indicates that there was a negative correlation between the proposed model and the BIS, 1 indicates that there is a high correlation between the EEG channels and the BIS, and 0 refers to the absence of relation between them. As shown in Fig. 7, the features extracted from Ch2 were high correlated with the BIS index compared with Ch1. As a result, the features from Ch2 were used to design the proposed model. EEG signals from Ch2 were decomposed into four levels and HDE were calculated.

### Model performance

In this study, the DoA was predicted, and the obtained results were compared with BIS values. The EEG signals were used as inputs data to the proposed model. EEG signals were decomposed into four levels. The HDE features were extracted from each level, then the extracted features were sent into the LS-SVM. The EEG data was divided into the training and testing sets. Table 2 presents our findings on DoA prediction. All results from 18 subjects were recorded, and the average of accuracy was calculated and considered in this experiment. The obtained results showed that the features from level 2 gave better prediction results compared to levels 1, 3, and 4. The best accuracy result was achieved with Awake state. The proposed model recorded 94% with moderate anaesthesia state. The maximum accuracy was achieved when two parameters γ and σ associated of the LS-SVM were set as γ = 1 and σ = 1.

**Table 2:** The prediction results of the proposed model

Anaesthetic states	Accuracy based on HDE features extracted from four levels (%)			
	Level	Level 2	Level 3	Level 4
Awake	89	96	82	81
Moderated anaesthesia	83	95	79	77
Deep anaesthesia	78	94.5	73	70

**Fig. 8:**
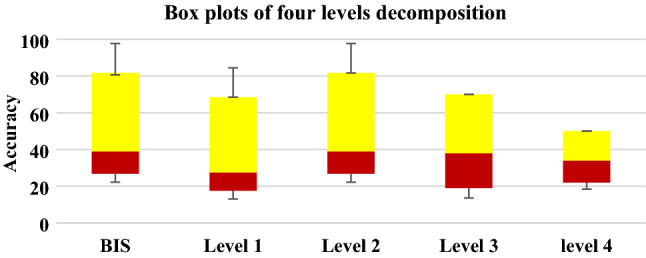
Flowchart of HDE produced from four layers

To investigate the effectiveness of the HDE features on the DoA, box plots were used. Figure 8 shows the HDF features extracted from 4 levels. It can be noticed that HDE features from level 2 are highly correlated with the BIS. The results in Fig. 8 were compatible with our findings in Table 2.

**Fig. 9:**
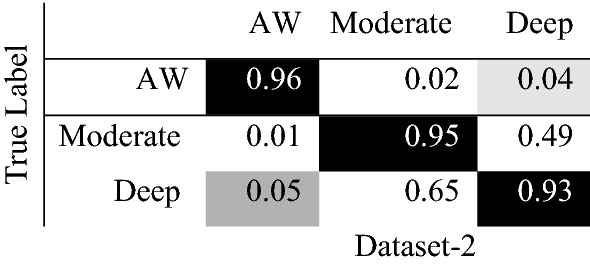
Confusion matrix of the proposed model

In order to verify the performance of the proposed model, the confusion matrices were calculated. The confusion matrix presents the False Negatives, True Positives, False Positive and True Negatives of anesthesia states. Figure 9 shows the confusion matrix of the proposed model, we can notice that the true positive of the AW state was the highest compared with other states. However, deep anesthetic state was the lowest number of true positive predictions. Our findings showed that some classes of depth anesthesia were classified as moderate anesthesia.

### Model interpretation

#### Performance evaluation based on regression model

**Fig. 10:**
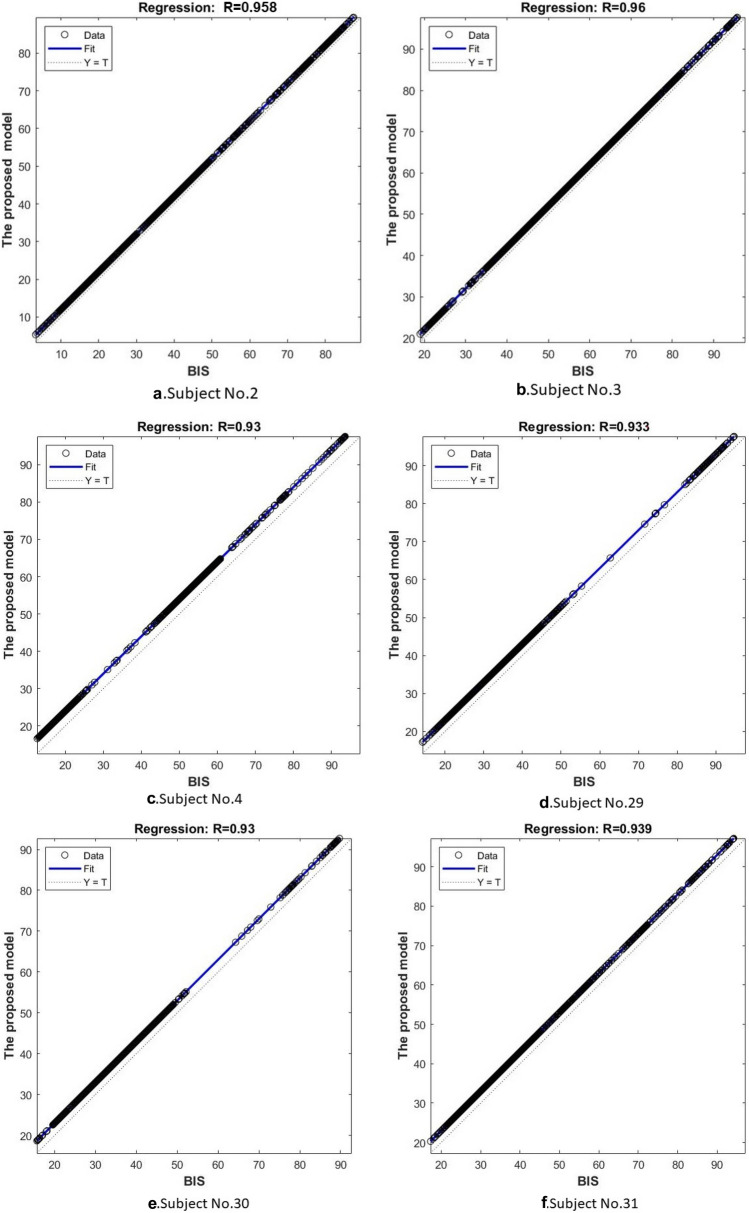
Regression line for the BIS index and proposed model

**Fig. 11:**
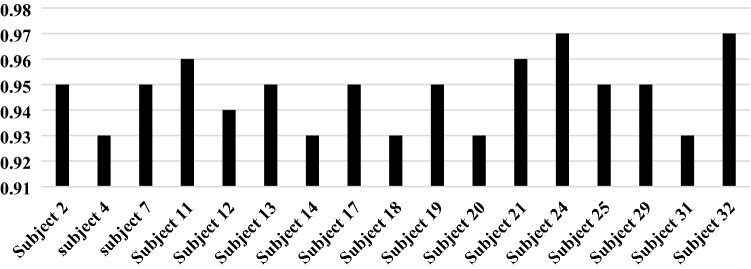
Coefficient determination for all subjects

In this experiment, we used a regression metric, which is also called coefficient determination, to determines the degree of similarity between the proposed model and the BIS. The regression was calculated for all the 22 participants, and its values were recorded. The coefficient determination value ranges from 0 to 1. If the output of regression is close to 1, it means that the proposed model had a strong relationship with the BIS index, and vice versa. The regression line of the proposed model and the BIS for subjects 2,3,4,29,30,31 was shown in Fig. 10. From the obtained results, it can be noticed that the proposed model and the BIS index are correlated with a high agreement. Figure 10 reports the coefficient determination for all subjects conducted in this study. The average of the coefficient determination for the 22 subjects was 0.96.5. The obtained findings in Figs. 10 and 11 proved that the proposed model and BIS index had the same behavior.

#### Performance evaluation based on Quantile–Quantile (Q-Q) plot

**Fig. 12:**
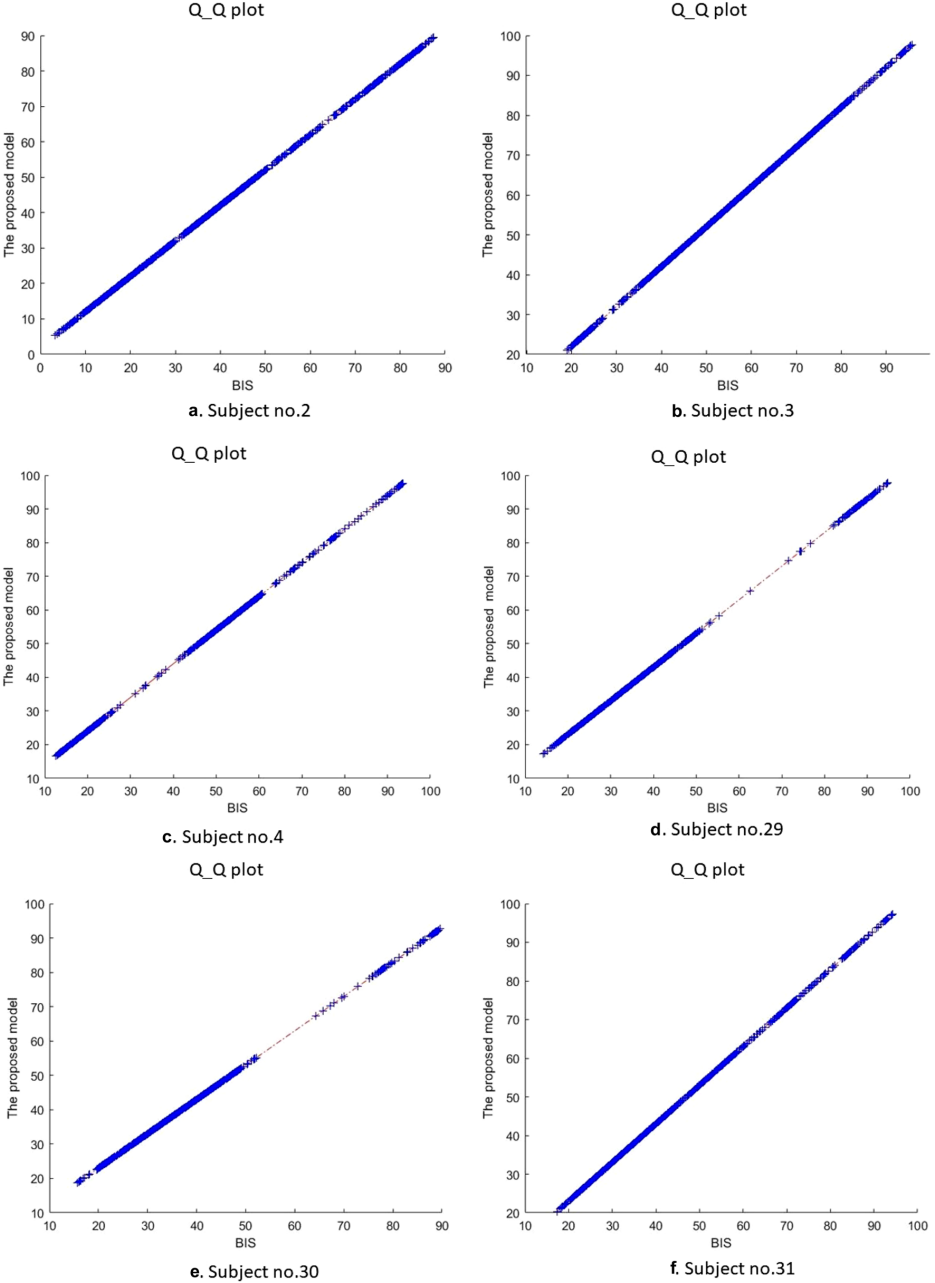
Q_Q plot between BIS index and proposed model

The Q-Q plot is a graphic approach which is used to determine the validity of two samples based on their distributions, and behaviors. In this experiment, we adopted a Q-Q plot to measure the degree of similarity between the proposed model and the BIS index based on their distributions. The quantiles of the prosed model were plotted against the BIS index's quantiles. The quantiles refer to percent of points that lie below a given value. Based on Q-Q plot, about 30% of the datapoints should fall below the given value while the rest of the datapoints should lie above that value. A 45-degree reference line was plotted. If our model and the BIS index have the same distribution and behaviors, all points must fall approximately along this reference line. Figure 12 shows the Q-Q plot of the proposed model and the BIS index for six subjects.

#### Performance assessment based on Pearson correlation coefficient

## Discussion

In all, we obtained good prediction results in the DoA using EEG signals. In this research we performed an analysis of EEG signals using different statistical metrics, to predict the depth of anesthesia (DoA).

**Fig. 13:**
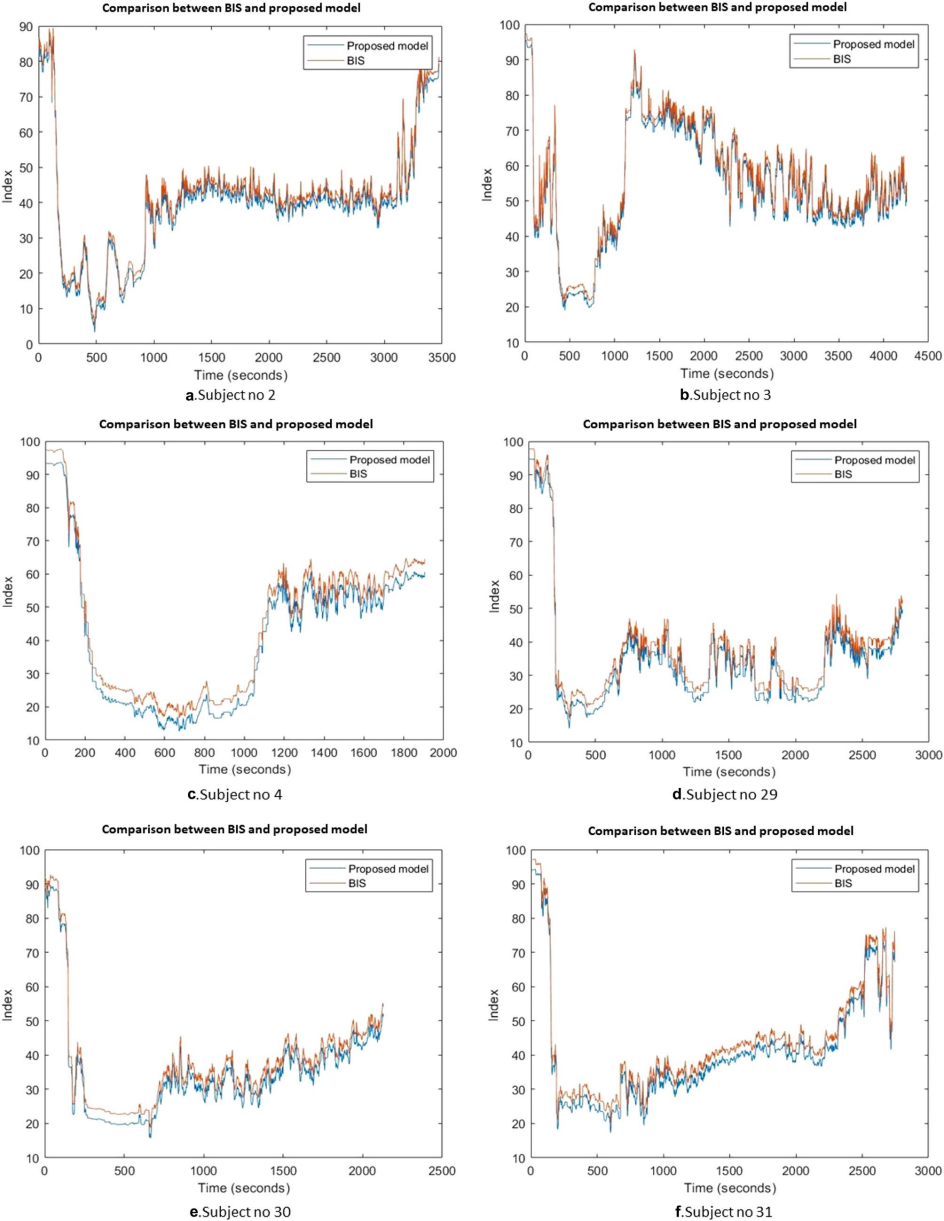
Comparison between BIS index and proposed

**Fig. 14:**
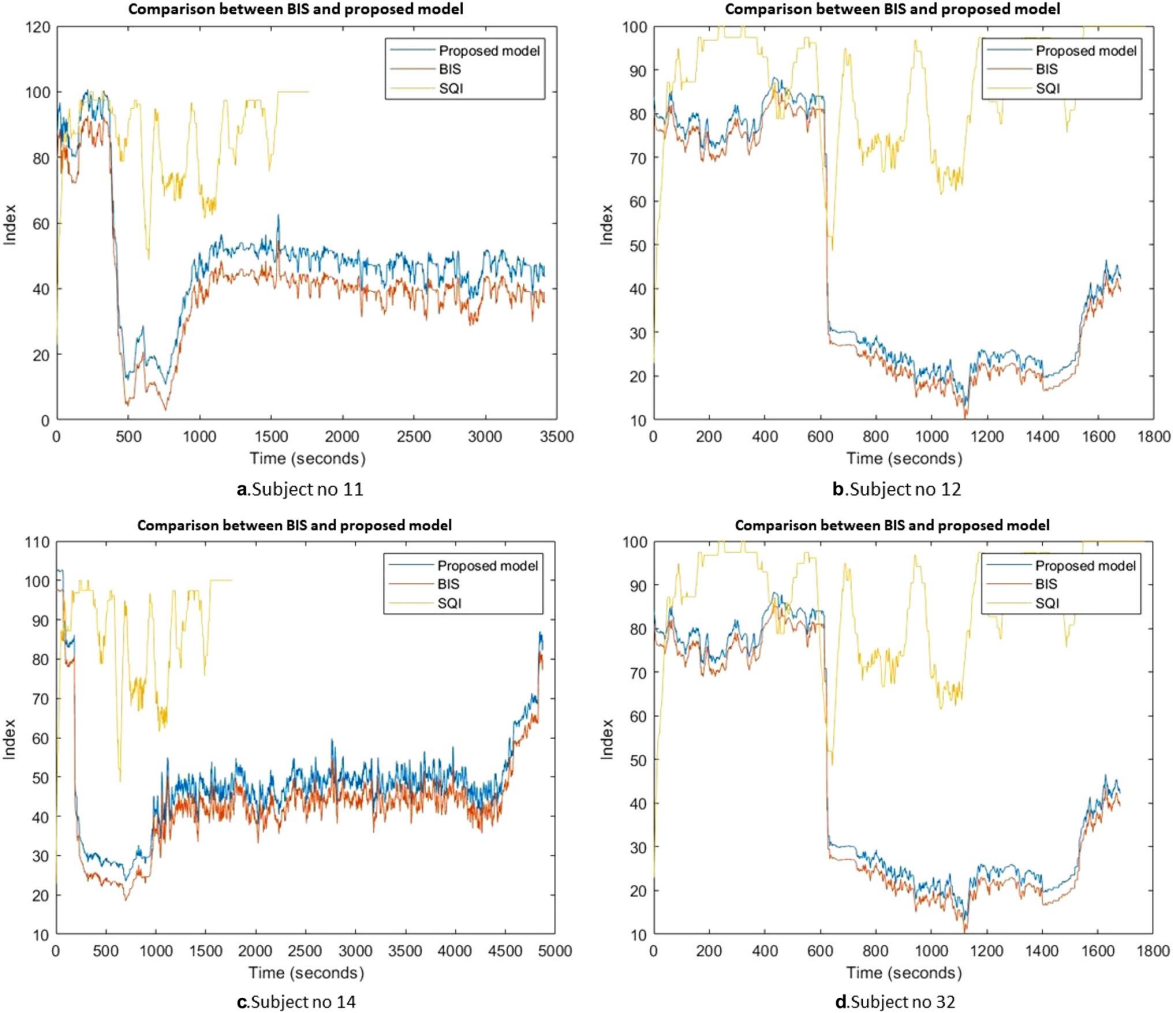
Comparison between BIS and proposed model in case of power signal quality

1. A comparison with previous studies was conducted. Table 3 reports the comparison results. Sadrawi et al., [48] predicted the DoA using an artificial neural network based on EMD. In that study, EEG signals were combined with EMG, SBP, HR, pulse, and DBP to predict the DoA. They found that artificial neural networks recorded a lower error than BIS. However, authors did not test their model in the case of low signal quality which is considered an essential test for any DoA model. However, in our study, the proposed model was tested in the case of poor signal quality. Liu et al., [47] recorded an accuracy of 93.5%. Short-time Fourier transform coupled with deep convolutional neural network CNN model was applied to EEG signal to predict the DoA. The BIS values were used as an index to assess the proposed model. Comparing with our results, it can be noticed that the obtained accuracy by our model was around 2% higher than Liu et al., [47]. Chowdhury et al., [23] adopted an image based deep convolutional neural networks approach to predict the DoA. An average accuracy of 86% was obtained in that study. Chowdhury et al., [23] recorded less accuracy than our method. In our previous study, Diykh et al. [17] a wavelet-based Fourier transform model was applied to monitor the DoA. In both studies Diykh et al. [17] and [35] the same dataset of our paper was used. However, in this study, we used more subjects to test our method, and used different metrics to evaluate our method. Based on the obtained results, the proposed model predicted the DoA accurately compared with the previous models.
2. The correlation coefficient was employed to assess the relationship between the BIS and the proposed model. Figure 6 showed the comparisons between the proposed model and the BIS index for subjects 2, 3, 4, 29, 30, and 31. Based on the results in Fig. 13, we can notice that the proposed model produced a similar behavior to the BIS. The obtained results demonstrated that the HDE features were very close to the BIS values. The values of the correlation coefficients for the 22 subjects were calculated. The average of correlation coefficients' average was 95.5%. The results in Fig. 13 proved that the proposed model produced a good performance across all subjects.
3. In this research, the proposed model's performance was assessed in the case of the power signal quality indicator. The EEG recordings were taken from four patients’ IDs (11,12,14,32) where the BIS failed to reflect the DOA. Figure 14 reports the obtained results for four subjects’ IDs(11, ID.12, ID.14, and ID.32). From the obtained results in Fig. 14a for subject ID 14, we can observe that the BIS index failed to reflect its values from 500 to 900 s, where the proposed model assessed the DoA accurately. Another case in which the BIS was not capable of showing the DoA was indicated in Fig. 14.b from the 800 s to the 1100 s, where the proposed model successfully predicted the DoA. For subject ID 11 and ID 12, the BIS failed also to reflect the patient’s state from 550 to 750 s, and from 800 to 1100 s; at the same time the proposed model was more accurate and consistent to show the changes from one anesthetic state to another.
4. The HDE was compared with different entropy features including sample entropy (MSE), multiscale fuzzy entropy (MFE), hierarchical fuzzy entropy (HFE), and hierarchical entropy (HE) which were used in the comparisons. Table 4 reports the average of coefficient determination, and AUROC of the HDE with MSE, MFE, HFE and HE. The HDE obtained 96%, and 95% while MSE, MFE, HFE and HE achieved less accuracy. It was evident that the HDE was more effective in the DoA than MSE, MFE, HFE and HE.

**Table 3:** Comparisons among the proposed model with previous studies

Authors	Approach	Signal	Accuracy
Liu et al., [47]	short-time Fourier transform coupled with deep convolutional neural network	EEG	93%
Sadrawi et al., [48]	Empirical mode decomposition integrated with artificial neural networks	EEG, EMG, HR, pulse, SBP, DBP	MAE of 6.54 with 6.69 of standard deviation
Diykh et al., [17]	Wavelet technique based on Fourier transform	EEG	
Diykh et al., [35]	Complex network-based spectrum technique	EEG 86	
Chowdhury et al., [23]	Image based deep convolutional neural network model	ECG and PPG signals	86%
The proposed model	HDE	EEG	95%

**Table 4:** Comparisons among HDE, with different entropy features

Technique	Coefficient determination	AUROC
MSE	88.2	87.1
HE	85.6	84.7
MFE	83.4	84.6
HFE	78.6	77.3
HDE	96.45	95.25

## Conclusions

In this study, a robust model was proposed to predict the DoA using HDE. The proposed model was assessed using several statistical metrics. The proposed model provides two contributions in terms of algorithm applicability. First, the proposed model serves as an efficient DoA monitor because it calculates the BIS value by taking only one channel of EEG as an input. This feature makes it possible to calculate BIS with a relatively simple procedure, compared with previous methods. Second, the extracted features are based on HDE, which is used to monitor the DoA, and can provide a deeper understanding of EEG mechanisms during the DoA. Further investigations can be made to clinical settings where the consciousness level must be monitored. However, our research has some limitations that require additional research. The data used in the performance evaluation may not have been sufficient to generalise the extracted features between EEGs and the BIS index. Additional research is needed to evaluate the proposed model using EEG recordings collected from various surgeries at multiple hospitals. Hence, the proposed model can be utilised as a low cost, and a simple model to predict the DoA using single channel EEG signals.
